# The Gender Violence - Implicit Association Test to measure attitudes toward intimate partner violence against women

**DOI:** 10.1186/s41155-020-00165-6

**Published:** 2020-11-10

**Authors:** Victoria A. Ferrer-Perez, Andrés Sánchez-Prada, Carmen Delgado-Álvarez, Esperanza Bosch-Fiol

**Affiliations:** 1 Faculty of Psychology, University of Balearic Islands, Ctra. Valldemossa km, 7’5, 07122 Palma de Mallorca, Spain; 2 Faculty of Psychology, Pontifical University of Salamanca, C/Compañía, 1-5, 37002 Salamanca, Spain

**Keywords:** Intimate partner violence against women, Attitudes, Implicit Association Test (IAT), Gender Violence - Implicit Association Test (GV-IAT)

## Abstract

Attitudes play a central role in intimate partner violence against women and are related to its origin, to the responses of women who suffer violence, and to the settings where it occurs. In fact, these attitudes are recognized as one of the risk factors linked to violent perpetration and to public, professional, and victim responses to this type of violence. However, even though available research generally shows a broad rejection of this violence, it remains a serious social and health problem that has reached epidemic proportions. This suggests that the information available about these attitudes (obtained through explicit and direct measures, i.e., self-reports) may be distorted or influenced by factors such as social desirability. In this context, the overall objective of our research project is to provide multi-method measures (explicit and implicit) of attitudes toward intimate partner violence against women, and the main goal of this paper is to propose an instrument for the implicit measurement of these attitudes. In this regard, the Implicit Association Test (IAT) is the most common procedure used, providing a superior predictive validity compared to explicit measures for socially sensitive topics. We will present an exploratory study that describes its adaptation for our purposes, and the development of the Gender Violence - Implicit Association Test (GV-IAT) to use among Spanish-speaking populations, and discuss the strengths and limitations of this proposal.

## Introduction

Violence against women and girls (VAW) is a gender-based violence recognized by the international community as a fundamental violation of human rights and as a social and public health problem of epidemic proportions, which can take multiple forms and occurs in different contexts (Devries et al., 2013; European Union Agency for Fundamental Rights, 2014; Stockl et al., 2013; World Health Organization, 2013). One of the most common forms of VAW is that which is inflicted by a male partner, referred to as intimate partner violence against women (IPVAW) (World Health Organization, 2013). This violence comprises a whole range of coercive sexual, psychological, and physical aggression acts, inflicted against adult or teenage women by their current or previous partner (United Nations, 2006).

Attitudes can play a central role in VAW and IPVAW (Flood & Pease, 2009; Gracia & Lila, 2015; Gracia, Rodríguez, & Lila, 2015; Gracia & Tomás, 2014; Heise & Kotsadam, 2015; Wang, 2016), both in terms of the origin of that violence, including the community where it occurs, and in the responses of women who suffer it (Capaldi, Knoble, Shortt, & Kim, 2012; Copp, Giordano, Longmore, & Manning, 2019; Fulu, Jewkes, & Garcia-Moreno, 2013; Gracia, Garcia, & Lila, 2014; Jewkes, Flood, & Lang, 2015; Puente, Ubillos, Echeburua, & Paez, 2016). In fact, some of the main risk factors for the occurrence of this type of violence are acceptance, in addition to tolerant beliefs and attitudes toward this violence, all at sociocultural and individual levels (Ferrer & Bosch, 2014; Garcia-Moreno et al., 2015; Gracia & Tomás, 2014; Puente et al., 2016). This work is centered on the analysis of social attitudes (public opinion or public attitudes) towards IPVAW.

In countries such as Spain, the measurement of explicit attitudes toward IPVAW demonstrate that around 90–95% of public opinion rejects this violence (Ferrer & Bosch, 2014; Meil, 2014; Spanish Government Office for Gender-Based Violence, 2014; Spanish Ministry of Health, Social Services and Equality, 2013), even though available data indicate that between 11 and 25% of women have suffered IPVAW at some point in their life and between 3 and 15% have experienced it in the last year (European Union Agency for Fundamental Rights, 2014; Spanish Ministry of Health, Social Services and Equality, 2015). This discrepancy between informed attitudes and the reality of the IPVAW may be due, among other things, to the social desirability effect in explicit measures (Eckhardt, Samper, Suhr, & Holtzworth-Munroe, 2012; Gracia et al., 2015; Gracia & Lila, 2015). For example, some research points out that males and people with a traditional gender role would have more positive attitudes toward IPVAW than women and people with an equal gender role attitude (Abrahams, Jewkes, Laubscher, & Hoffman, 2006; Flood & Pease, 2009; Obeid, Chang, & Ginges, 2010; Sánchez-Prada, Delgado-Álvarez, Bosch-Fiol, & Ferrer-Pérez, 2018). However, in sensitive areas such as this, respondents may avoid sharing their real beliefs because of a fear of negative consequences or judgments and may instead offer distorted responses, provide inaccurate information, or present themselves in a more socially acceptable manner (Eckhardt et al., 2012; Gracia et al., 2015).

In summary, the availability of reliable and valid measures of the acceptability of IPVAW is important for research and intervention purposes, as they can provide knowledge about the social conditions that contribute to them (Gracia et al., 2015; Gracia & Lila, 2015) and because explicit measures do not always achieve this goal.

### The measure of attitudes

The nature of attitudes can be analyzed from different points of view: in dual-attitude models, the main postulate is that implicit and explicit attitudes have separate mental representations (Petty, Briñol, & DeMarree, 2007), while single-attitude models imply that there is only a single construct of attitude and individual differences in the tendency to like or dislike an attitude object across different situations (Fazio & Olson, 2014).

Nevertheless, and beyond any discussion about its nature or composition, one of the fundamental topics in attitudinal studies is the distinction between explicit and implicit approaches to assess them (Gawronski & Bodenhausen, 2006). In general, the measurement of attitudes through explicit measures and direct procedures, such as self-reports, have been successful due to their ease of administration, cost-effectiveness, and efficacy (Paulhus & Vazire, 2007). However, these procedures entail substantive or methodological limitations. In fact, some researchers have questioned the validity of self-report measures, arguing that in the case of IPVAW, social desirability and self-presentational concerns produce inaccuracy (De Houwer, Teige-Mocigemba, Spruyt, & Moors, 2009; Fazio & Olson, 2003; Nosek, 2005; Nosek, Hawkins, & Frazier, 2011; Ryan, 2013; Scott & Straus, 2007), supporting the need for alternative methods of assessment.

For this reason, some researchers have used implicit methods of assessment that measure attitudes at an indirect level and can provide information that is distinct from self-reports and predicts social behavior (Nosek et al., 2011; Olson & Fazio, 2004). In fact, previous research indicates that implicit measures are widespread and robust on average (Nosek et al., 2007; Payne et al., 2010) and aggregate scores of these measures at the macro level (such as states or countries) show strong relations with indicators of discrimination at the same level of analysis (Leitner, Hehman, Ayduk, & Mendoza-Denton, 2016; Nosek et al., 2009).

In this sense, and in an attempt to overcome the limitations of direct measures, attitudes can be measured through reaction times (RT) (Blair, Dasgupta, & Glaser, 2015; Kihlstrom, 2004; Tosi, Ledesma, Poo, Montes, & López, 2018). One of the best researched implicit instruments is the IAT, developed by Greenwald, McGhee, and Schwartz (1998). In general terms, IAT is an indirect measure used to assess the relative strength of cognitive associations between two target concepts and an evaluative dimension by utilizing a number of response discrimination tasks (Fazio & Olson, 2003; Greenwald et al., 1998). Specifically, the IAT measures the strength of these associations (implicit attitudes) by comparing response latency (in milliseconds) to different pairings of the concepts of interest with target stimuli. The preferences of the individual are inferred from the speed of responding to stimuli in a categorization task (Bar-Anan & Nosek, 2014; Blair et al., 2015; De Houwer et al., 2009; Greenwald, Nosek, & Banaji, 2003; Nosek, Greenwald, & Banaji, 2007).

It is worth noting that IAT is increasingly used for analysis in several areas of social cognition and social phenomena (Payne & Gawronski, 2010) such as prejudice (McConnell & Liebold, 2001), self-esteem (Greenwald & Farnham, 2000), or social identity (Greenwald, Banaji, Rudman, Farnham, & Nosek, 2002). In fact, the IAT has quickly become the most frequently chosen implicit measurement tool for many relevant social, cognitive, and personality variables (Nosek, Bar-Anan, Sriram, Axt, & Greenwald, 2014; Olson & Fazio, 2004). The paper published by Greenwald et al. (1998) is one of the most influential in personality and social psychology, having had an even greater impact outside psychology, as indicated by the millions of visitors to the IAT website (Schimmack, 2019).

Despite this popularity, it must be pointed out that there are different controversies about the internal structure of the IAT, the nature of the underlying processes, the possible effects of confounding variables, and, particularly, about their validity (Nosek, Greenwald, & Banaji, 2007; Rezaei, 2011; Schimmack, 2019; Tosi et al., 2018). One of the most important controversies concerns precisely the validity of what IAT measures. From a dual-attitude model, some researchers consider that it measures implicit constructs or unconscious processes, such as implicit attitudes or implicit preferences that cannot be measured with self-report measures (Banaji & Greenwald, 2013; Greenwald et al., 1998; Nosek, Greenwald, & Banaji, 2007). However, others argue that it is an implicit or indirect measure of the same constructs that are measured with explicit measures (Samayoa & Fazio, 2017), suggesting that it is better to avoid writing about implicit constructs (Greenwald & Banaji, 2017; Schimmack, 2019). Another controversy arises from the fact that the discrepancy between implicit and explicit measures is more often the standard than the exception. In fact, correlations between implicit and explicit measures tend to be low (Hofmann, Gawronski, Gschwendner, Le, & Schmitt, 2005; Kurdi & Banaji, 2019), to the extent that some authors question whether this is a convergent or a discriminating validity criterion (Tosi et al., 2018). In this sense, the debate is still open on how correlations with explicit measures of the same construct should be interpreted and which results would support greater evidence of validity: high correlations would support the idea of convergent validity, while low correlations would support the idea of discriminant validity. This divergence in interpretation, as well as the variability of explicit-implicit correlations as a function of the measured construct, supports the need for greater empirical evidence to advance the construction of a theoretical interpretative framework for the meaning of IAT measures. A detailed discussion about the limitations of the IAT procedure is addressed in Schimmack (2019) or Tosi et al. (2018).

### Applying implicit measures to assess attitudes toward intimate partner violence against women

Most studies examining public attitudes toward IPVAW have not included both implicit and explicit measures jointly (Sánchez-Prada et al., 2018). Certainly, implicit measures, such as the IAT, have been used to assess attitudes toward rape (Süssenbach, Albrechet, & Bohner, 2017), sexual violence (Larue et al., 2014), or dating violence (Lee, Begun, DePrince, & Chu, 2016), or to predict violent behaviors of some types of violent perpetrators (Bluemke et al., 2017; Simane-Vigante, Plotka, & Blumenau, 2015).

But only a few previous studies have analyzed attitudes toward IPVAW using implicit measures, such as the IAT: Robertson and Murachver (2007) examined offenders’ attitudes toward violence using an IAT measure in participants that were both incarcerated for intimate partner violence and nonincarcerated. The attitudes toward violence were more similar in both groups when measured explicitly, but if measured by IAT, the incarcerated sample had significantly more positive attitudes toward violence. Eckhardt et al. (2012) used three IAT measures to examine attitudes toward women, violence, and the associations between gender and violence in a sample of men from a batterer intervention program and nonviolent men from the community. Measured by IAT, offenders had significantly more positive attitudes toward violence, and stronger associations between women and violence. But no significant differences were found between groups on the explicit attitude measurement. They hypothesize that the explicit measures to assess attitudes toward IPVAW among batterers may be limited by their tendency to deny or minimize their violent behavior. Cantera and Blanch (2010) and Cantera and Gamero (2012) used the IAT to assess the strength of associations between some gender stereotypes (men as providers and women as caregivers) and violence (violent men and peaceful women), in the context of the debate about the benefits and limitations of a gender approach to understanding IPVAW.

To our knowledge, no previous studies have used the IAT specifically to assess implicit attitudes toward IPVAW, which is why we conducted a preliminary study (Sánchez-Prada et al., 2018) where subjects from two Spanish communities undertook an IAT. The results obtained supported the hypothesis that implicit measures of public attitudes toward IPVAW will enable better evaluations by neutralizing social desirability effects and the response control of subjects. Specifically, as a result of this preliminary study, and assuming that IAT is an implicit measure of the same constructs that are measured with explicit measures (Samayoa & Fazio, 2017), we propose a form of personalized IAT to this aim: the GV-IAT primarily addressed to Spanish-speaking populations where this procedure is scarcely used (Tosi et al., 2018).

## Method

The present paper aims to focus on the use of the Implicit Association Test (IAT), developed by Greenwald et al. (1998), to assess social attitudes (public opinion) towards IPVAW and to present an exploratory study with results that permit us to propose a form of personalized IAT to this specific subject: the Gender Violence - Implicit Association Test (GV-IAT).

### Participants

In an exploratory study, the GV-IAT was applied to a convenience sample of 89 psychology students from two Spanish universities: 10 men (11.2%) and 79 women (88.8%), with an average age of 19.65 years (SD = 4.53).

### Materials

The *Inventory of Distorted Thoughts about Women and Violence* (IPDMV in the Spanish acronym, Echeburua & Fernández-Montalvo, 1998; adapted version of Ferrer, Bosch, Ramis, Torrens, & Navarro, 2006) is a 24-item scale with a four-point response scale and four dimensions: inferiority of women compared to men (7-items, *α* = .88), blaming female victims of abuse (8-items, *α* = .66), violence as an appropriate problem-solving strategy (5-items, *α* = .70), and minimization of IPVAW as a problem and exoneration of the abuser (4-items, *α* = .52). Higher scores indicate higher levels of distorted thoughts about women and violence.

The *Inventory of Beliefs about Wife Beating* (IBWB, Saunders, Lynch, Grayson, & Linz, 1987; Spanish version of Expósito & Ruiz, 2010), a 30-item scale designed to assess participants’ explicit attitudes toward wife-beating, which comprises five dimensions: wife-beating is justified (12 items, *α* = .86), wife gains from beating (7 items, *α* = .78), help should be given (5 items, *α* = .73), offender should be punished (3 items, *α* = .61), and the offender is responsible (4 items, *α* = .62).

*The Gender Violence Implicit Association Test (GV-IAT.* Although “the most common version of the IAT is the one originally introduced by Greenwald et al. (1998), the traditional IAT” (McConnell & Rydell, 2019, p. 151), there are some variants or forms of personalized IAT, with some modifications to the original (see McConnell & Rydell, 2019; or Olson & Fazio, 2004). Hereafter, we present the GV-IAT, an IAT developed and adapted to measure implicit attitudes towards IPVAW.

The GV-IAT, like any form of IAT, consists of asking participants to classify target stimuli, presented in the center of a computer screen, into two response categories (target concepts and attribute concepts). Each of these categories, located on the left and right side of the screen, is represented by two different concepts. Participants have to press a computer key (left or right) as quickly as possible to classify the word in the center (target stimuli) into one of the two categories located on the sides, creating compatible and incompatible pairings.

Specifically, the GV-IAT is a form of personalized IAT, an implicit measure of public attitudes toward IPVAW. That is why the concepts of the target category are *Gender violence* vs. *Non gender violence* and the concepts of the attribute category are *Good* vs. *Bad*. Six words from each of the aforementioned categories were used as stimuli: (a) Attack, Force, Humiliate, Hit, Torture, and Infringe for the category Gender Violence; (b) Support, Collaborate, Cooperate, Empathize, Respect, and Tolerate for the category Non gender violence; (c) Wonderful, Excellent, Phenomenal, Best, Positive, and Optimum for the category Good; (d) Horrible, Terrible, Disastrous, Worst, Negative, and Appalling for the category Bad. This English translation is shown in order to facilitate comprehension; however, the GV-IAT is mainly addressed to Spanish-speaking populations, which is why the IAT attribute categories are given in Spanish (see Appendix)

In the case of the target category (Gender violence vs. Non gender violence), it is necessary to make two remarks: First, it is important to note that under Spanish law (Organic Act 1/2004, of 28 December, on Integrated Protection Measures against Gender Violence 2004), IPVAW is known as gender violence (see Ferrer & Bosch, 2014), which is why GV-IAT uses gender violence to refer to IPVAW and also to define the target category. Secondly, the target stimuli were selected from the *Toughness and Tenderness Scale* (Cantera & Blanch, 2010), applied by the authors in previous Spanish research using IAT in the study of some aspects of IPVAW. Specifically, ten expert members of the Gender Studies research group of our university individually indicated those words on the scale that best defined the two concepts of the category. The words with the highest level of agreement were selected for the study. The inter-judge reliability of the stimulus matching scores (ranged from 1 to 7) was estimated by an intraclass correlation index (Shrout & Fleiss, 1979). This method is the most appropriate statistical index for estimating the reliability of the evaluations by expert judges when they are expressed on a quantitative scale (Fleiss & Cohen, 1973). This index estimates the agreement or equivalence of the scores given by the judges to the different stimuli, whereby a value ≥ .75 indicates an excellent agreement or reliability (Fleiss, 1986). The value obtained for our stimuli was .999 by the absolute agreement method, which indicates an excellent coincidence in the scores issued by the judges. Additionally, there are different techniques to ensure the reliability of the evaluations issued by the judges. The Aiken V index (Aiken, 1985) has the advantage that it quantifies the relevance of each item or stimulus valued by the judges, taking into account the mean, as well as the range and the lowest value of the evaluation (García-Sedeño & García-Tejera, 2013). An additional advantage of this index is that its interpretation is based on the statistical significance obtained from the tables of critical values (Aiken, 1985). Thus, for a significance level *α* = .01, the stimuli with Aiken V values lower than .75 should be eliminated (Charter, 2003). The values obtained in our study were optimal for all stimuli (.98 for Tolerate and 1.0 for all others).

Additionally, 588 university students, who volunteered to participate in a previous pilot study, completed the *Toughness and Tenderness Scale* (Cantera & Blanch, 2010), in which they rated a total of 48 words as a possible expression of gender violence in the context of a heterosexual intimate partner relationship, on a scale from 1 (“it’s not gender violence”) to 4 (“it is gender violence”). A subsequent repeated measure ANOVA with data obtained (*F* (47, 511) = 4858.649, *p* < .001) confirmed the appropriateness of the stimuli, since there were significant differences between among each of the six words selected for the category *Gender violence* and each of the six words selected for the category *Non gender violence* (*p* < .001 for all of the pairwise comparisons applying Bonferroni’s correction).

For the attribute category, we use the concepts *Good* vs. *Bad* following previous studies (Eckhardt et al., 2012; Süssenbach et al., 2017), and the stimuli were selected following the proposals of the Spanish version of the Harvard Project Implicit (https://implicit.harvard.edu/implicit/spain/) and the study by Briñol, Horcajo, Becerra, Falces, and Sierra (2002).

In line with the proposal of Greenwald et al. (2003), participants completed the GV-IAT task in seven blocks, each of which came in two versions: Of the seven blocks, three were considered practice trials (B1-B2 included 24 trials; B5 included 48 trials), and four were the critical blocks (B3-B6 included 24 trials; B4-B7 included 48 trials), where a trial is deemed to be the time from when the target appears onscreen until the stimulus is correctly categorized. Regarding the versions, version 1 starts with the compatible critical phase (pairing the categories Gender-Violence + Bad on one response key and Non gender violence + Good on the other) (Table 1), and version 2 with the incompatible critical phase (pairing the categories Gender-Violence + Good on one response key and Non gender violence + Bad on the other) (Table 2).

**Table 1:** Sequence of stimuli presentation initiating the IAT with the compatible critical phase

Blocks	Trials	Phase	Categories assigned to the left key (S)	Categories assigned to the right key (L)
B1	24	Practice	Non gender violence	Gender violence
B2	24	Practice	Good	Bad
B3	24	Critical compatible	Non gender violence	Gender violence/Bad
B4	48	Critical compatible	Non gender violence	Gender violence/Bad
B5	48	Practice	Gender violence	Non gender violence
B6	24	Critical incompatible	Gender violence/Good	Non gender violence/Bad
B7	48	Critical incompatible	Gender violence/Good	Non gender violence/Bad

**Table 2:** Sequence of stimuli presentation initiating the IAT with the incompatible critical phase

Blocks	Trials	Phase	Categories assigned to the left key (S)	Categories assigned to the right key (L)
B1	24	Practice	Gender violence	Non gender violence
B2	24	Practice	Good	Bad
B3	24	Critical incompatible	Gender violence/Good	Non gender violence/Bad
B4	48	Critical incompatible	Gender violence/Good	Non gender violence/Bad
B5	48	Practice	Gender violence	Non gender violence
B6	24	Critical compatible	Non gender violence/Good	Gender violence/Bad
B7	48	Critical compatible	Non gender violence /Good	Gender violence/Bad

At the beginning of the GV-IAT, instructions (obtained from the Project Implicit of Harvard University in its Spanish version: https://implicit.harvard.edu/implicit/spain/) are given for participants to complete blocks 1 and 2: subjects must classify words into groups as fast as they can, committing the minimum number of errors; the list of words (stimuli) is displayed onscreen with an explanation to use the letter S (for words that belong to the category on the left of the screen) or L (for the words in the category on the right). Participants are also instructed that if they make an error, a red X will appear and they can then mark the correct answer. Once the first two test blocks have been completed, and before starting the next ones, the following instructions appear onscreen: *now the four categories appear together and each word belongs to a single category, press the letter S when the words belong to the category on the left of the screen and letter L when the words belong to the category on the right; green and white labels help to identify the correct category* (this is an English summary of the original Spanish instructions).

### Procedure

The GV-IAT is designed to be applied in individualized sessions lasting approximately 10 min, where each subject may perform the test by typing on a desktop computer located inside a booth isolated from outside noise (e.g., a lab in the university).

Half of the participants will be randomly assigned to one of the two versions of the GV-IAT (version 1 or version 2). Consequently, the presentation order for stimuli will be randomly controlled across participants, and the position on the screen of target categories will be counterbalanced (half to the left; half to the right). In both cases, each subject will undertake a total of 240 trials.

The counterbalanced stimuli in each block appear onscreen, and in each trial, the participants receive immediate feedback of their response and are forced to enter the correct answer in order to continue (forced-choice task). In this respect, it is necessary to point out that, according to the literature, there are two IAT procedures (Nosek et al., 2014; Nosek, Greenwald, & Banaji, 2007): the typical IAT procedure (Greenwald et al., 1998), which includes the provision of immediate feedback when the participant has made an error, at which point the user is forced to enter the correct choice before advancing to the next trial (with feedback); and the personalized IAT procedure (Olson & Fazio, 2004), which did not include error feedback, allowing the IAT to go on, even after an erroneous response, with no correction required (i.e., without feedback). Preliminary research (Sánchez-Prada et al., 2018) provides evidence that the best way to apply the GV-IAT is the forced-choice task or with feedback.

For this reason, it is very important that the stimulus clearly represents the category, so that it generates little doubt for the subject and the results maintain validity. Therefore, if the choice is incorrect, a red X appears and participants must mark the correct answer (if not, the program does not move on), and if the choice is correct, the program goes on to the next stimulus.

In order to facilitate correct performance of the experiment, it is necessary to clarify that the concepts of the target category (Gender violence vs. Non gender violence), as well as the stimuli that describe that category, appear on the computer screen in white letters, while the concepts of the attribute category (Good vs. Bad) and its associated stimulus words appear on the computer screen in green letters.

The OpenSesame (version 3.1.6, Mathôt, Schreij, & Theeuwes, 2012) computer program was used to design the IAT. Specifically, stimuli were displayed on a 20-in. screen with a PC running OpenSesame on Windows 8.

### Data analysis

The fundamental principle of the IAT is that where two concepts are strongly associated, and the response latency (RL) is less than when this is not the case. The IAT scores are calculated using a latency-based response obtained in the trials corresponding to compatible critical phase (RLc), and those obtained in the trials corresponding to incompatible critical phase (RLi), as per the order described. Based on those response latencies, and in order to analyze the IAT effect, a *D*-score is first calculated for each participant, according to the algorithm proposed by Greenwald et al. (Greenwald et al., 2003; Nosek, Greenwald, & Banaji, 2007), and subsequently optimized in several studies (Blanton, Jaccard, & Burrows, 2015; Fazio & Olson, 2003; Glashouwer, Smulders, de Jong, Roefs, & Wiers, 2013; Nosek et al., 2014; Nosek, Greenwald, & Banaji, 2007):

- Step 1: consider for analysis data from Blocks B3 and B4, and B6 and B7. In version 1, the blocks B3 and B4 correspond to the compatible phase (RLc), and the blocks B6 and B7 to the incompatible phase (RLi). In version 2, the blocks B3 and B4 correspond to the incompatible phase (RLi), and the blocks B6 and B7 to the compatible phase (RLc).
- Step 2: remove trials with latencies greater than 10,000 ms.
- Step 3: discard cases in which more than 10% of trials have latencies lower than 300 ms.
- Step 4: calculate the standard deviation for blocks B3 and B6 taken together, and the standard deviation for blocks B4 and B7.
- Step 5: calculate the means of the trials in each of the blocks B3, B4, B6, and B7.
- Step 6: calculate two difference scores (one between B3 and B6, and the other between B4 and B7), subtracting the means obtained in the compatible phase from their respective means in the incompatible phase (i.e., B6 – B3 and B7 – B4 in version 1; B3 – B6 and B4 – B7 in version 2).
- Step 7: each difference of means is divided by its corresponding standard deviation calculated in step 4.
- Step 8: average the two quotients obtained in step 7.

The theoretical basis behind this algorithm is that a shorter response time (RL) indicates greater automatic association between the categories presented (Banaji, 2001). This means that (a) people with rejection attitudes towards IPVAW will have a lower RL when associating theoretically compatible categories (i.e., Gender violence - Bad) than when associating theoretically incompatible categories (i.e., Gender violence - Good) and (b) people with acceptance attitudes towards IPVAW will not perceive as much cognitive dissonance between theoretically incompatible categories (i.e., Gender violence - Good) and will therefore more easily associate them with lower RLs in incompatible phases. The algorithm used estimates how much the RL of a person in an incompatible phase deviates from his own RL in a compatible phase (Dorantes, Ferrero, & Tortosa, 2014). The magnitude of this difference is considered an indicator of the degree of acceptance or rejection towards IPVAW: the greater the rejection, the greater the difference between the RL of the compatible and incompatible phases. In addition, the algorithm corrects for the effects of familiarity with the technique by not computing previous test blocks, which have the singular function of practicing the technique. Therefore, only the responses of the so-called critical phase are computed, which are recorded after a series of previous tests.

Regarding the topic of error latency treatment, there are two procedures recommended according to the literature: (a) replacing each error latency with the mean of the correct latencies for the respective block, adding a 600-ms error penalty (Nosek, Greenwald, & Banaji, 2007) and (b) integrating a “built-in penalty” in error latencies by computing the accumulated time of the wrong response and the time spent in correcting that first response (Greenwald et al., 2003; Nosek et al., 2014; Richetin, Costantini, Perugini, & Schönbrodt, 2015). Previous research (Sánchez-Prada et al., 2018) provides evidence that the best procedure for estimating the IAT effect in the case of the GV-IAT is the built-in error penalty algorithm.

The *D*-scores obtained are interpreted in a similar way to Cohen’s *d*. Thus, in GV-IAT, positive *D*-scores express a longer latency time when gender violence is associated with positive stimuli than when it is associated with negative stimuli. This indicates a perception of incongruence between positive stimuli and gender violence (i.e., RLi greater than RLc), and therefore, implicit rejection of gender violence: the higher the value of D, the stronger the rejection. In turn, values close to zero or negative *D*-scores express low differences in latency times, depending on whether gender violence appears to be associated with positive or negative stimuli, which indicates a perception of congruence between positive stimuli and gender violence (i.e., RLi similar or less than RLc), and therefore, the absence of rejection or implicit acceptance of gender violence. According to Cohen (1988), and in the same line proposed by Greenwald et al. (2003), we considered the rejection of gender violence (i.e., the intensity of the IAT effect) as null rejection if *D* < .20, as mild if .20 ≤ *D* < .50, as moderate if .50 ≤ *D* < .80; and strong if *D* ≥ .80.

## Results

The internal consistency of the GV-IAT was estimated following the split-half procedure recommended by Kurdi et al. (2019), through an online tool (available at https://bkurdi.shinyapps.io/reliCalc/). The average of the distribution of the 600 split-half correlations calculated by this procedure was .73, once a Spearman-Brown correction was applied for split-half reliability (Carpenter et al., 2019; De Houwer & De Bruycker, 2007).

Taking Cohen’s interpretation of the effect size magnitude as a reference (Cohen, 1988), the rejection of gender violence measured by the GV-IAT was distributed in this sample as follows: four cases (4.5%) with null rejection, 13 cases (14.6%) with mild rejection, 40 cases (44.9%) with moderate rejection, and 32 cases (36.0%) with strong rejection.

The explicit measures showed a strong explicit rejection of IPVAW, with scores close to the lower pole on a scale of 1 to 4, where the lower the score, the greater the rejection of GBV (*M*_IBWB_ = 1.52, SD_IBWB_ = 0.21; *M*_IPDMV_ = 1.60, SD_IPDMV_ = 0.18).

To compare the scores obtained with explicit (self-reported) and implicit (GV-IAT) measures of attitudes towards IPVAW, the sample was segmented into four groups with approximately 25% of the subjects in each case, based on the quartile scores on the GV-IAT. The results obtained are shown in Table 3.

**Table 3:** Preliminary results comparing GV-IAT and explicit measures

Group by D-quartiles	IAT effect (IPVAW rejection level)	IBWB^a^	IPDMV^b^
*D* < Q1 (*n* = 22)	*M* = 0.354; *SD* = 0.173 *D* range = -0.128–0.523 (null–mild rejection)	*M* = 1.57; *SD* = 0.255 (*n* = 20)	*M* = 1.65; *SD* = 0.194 (*n* = 20)
Q1 < *D* < Q2 (*n* = 22)	*M* = 0.631; *SD* = 0.057 *D* range = 0.525–0.700 (mild rejection)	*M* = 1.47; *SD* = 0.155 (*n* = 19)	*M* = 1.61; *SD* = 0.230 (*n* = 20)
Q2 < *D* < Q3 (*n* = 23)	*M* = 0.784; *SD* = 0.048 *D* range = 0.716–0.859 (moderate–strong rejection)	*M* = 1.45; *SD* = 0.173 (*n* = 19)	*M* = 1.56; *SD* = 0.172 (*n* = 21)
*D* > Q3 (*n* = 22)	*M* = 0.955; *SD* = 0.082 *D* range = 0.862–1.219 (strong rejection)	*M* = 1.57; *SD* = 0.223 (*n* = 21)	*M* = 1.60; *SD* = 0.185 (*n* = 21)

^a^Total score in the IBWB. Only 79 participants completed this scale

^b^Total score in the IPDMV. Only 82 participants completed this scale

As can be seen (Table 3), the four groups formed by segmenting the sample from the quartile scores on the GV-IAT differ in the level of rejection to IPVAW in relation to the cut-off points defined by Cohen (1988) and adapted by Greenwald et al. (2003), from null to strong rejection. Additionally, the scores obtained by these four groups on the two explicit measures were compared through an ANOVA and no statistically significant differences were detected either in the IBWB (*F*(3, 75) = 1.920, *p* = .134) or in the IPDMV (*F*(3, 78) = 0.909, *p* = .441), despite the fact that the degree of implicit acceptability/rejection of the IPVAW differed between these four groups (as shown by their GV-IAT punctuation). In fact, it could be noted that both explicit measures remain relatively constant and close to the “rejection of the IPDMV” pole (between *M* = 1.45 and *M* = 1.65, depending on the group), regardless of the different levels of acceptance-rejection detected with the GV-IAT (Table 3).

## Discussion and conclusions

As Garcia-Moreno et al. (2015) point out, the reduction of violence against women and IPVAW requires interventions from different sectors, and changes in individual and institutional discriminatory behaviors and attitudes. In this sense, a significant decrease in the violence against women and girls is achievable, but it requires sustained action to ensure that political commitments translate into meaningful change and support for coordinated, well-funded, evidence-informed strategies implemented by governments, communities, and civil society partners (Krisch, Eisner, Mikton, & Butchart, 2015). This support must be provided to activities that challenge discriminatory attitudes and behaviors toward women and girls, the tacit approval of violence against them, male control of female behavior, and constructs of masculinity that encourage male violence.

In this context, and because IPVAW is a sensitive area, there is a possibility that the respondents avoid sharing their real beliefs and provide inaccurate information, or socially desirable answers (Eckhardt et al., 2012; Gracia et al., 2015). As a result, the application, use, and interpretation of implicit measures is a relevant alternative because they have the potential to serve as useful methodological tools for testing hypotheses since they are guided by relevant theory and past literature (Fazio & Olson, 2003).

Indeed, the preliminary data obtained, and shown in this paper, have pointed out some strengths of the GV-IAT as a complementary measure to the explicit measures of attitudes toward IPVAW. Firstly, the main strength is that this is, to our knowledge, the first proposal of a personalized IAT that measures attitudes toward IPVAW and that has been designed specifically for Spanish-speaking people. Their use may allow the design of preventive actions and public policies to reduce this form of violence. Secondly, as occurs with all IAT forms, the GV-IAT does not require direct probing of subjects and may therefore reduce the impact of conscious intention or deliberate processes on responses and, consequently, the impact from desirable social effect (Kim, 2003; Nosek, Greenwald, & Banaji, 2007). Thirdly, despite the small size and homogeneity of the sample studied in this preliminary analysis, the GV-IAT internal consistency obtained is satisfactory, and it is in the range of .70–.90 usually reported in the literature on the IAT, demonstrating superiority in this aspect with regard to other implicit measures (Bar-Anan & Nosek, 2014; Gawronski & De Houwer, 2014; Nosek, Greenwald, & Banaji, 2007; Tosi et al., 2018). Finally, the discrepancies observed between implicit and explicit measures indicate that implicit measures would have a higher sensitivity to detect variability in the measured construct, in this case, attitudes towards IPVAW. Furthermore, according to the MODE model (Fazio, 1990; Fazio & Olson, 2014), these data would support the greater robustness, rather than immunity, to social desirability and deliberate misrepresentation of implicit measures, such as GV-IAT, compared to explicit measures (Gawronski & Hahn, 2019; Greenwald, Poehlman, Uhlmann, & Banaji, 2009; Kurdi et al., 2019).

Despite its strengths, this proposal also comes with a number of limitations and challenges. As aforementioned (Nosek, Greenwald, & Banaji, 2007; Rezaei, 2011; Schimmack, 2019; Tosi et al., 2018), the main limitation of the GV-IAT is the same that affects all implicit measures: the lack of direct procedures to obtain conclusive evidence on the validity of the construct (given the involvement of different sources of variation in the measurements obtained, the measurement artefact [responses before a computer screen], the mental processes involved in the responses, and the evolutionary variables that affect the interference of stimulus incongruence, and consequently, the latency times). Another limitation may be the discrepancy between implicit and explicit measures (shown by the low correlations obtained), normally found in research with both types of measurement tools (Hofmann et al., 2005; Kurdi & Banaji, 2019). This result supports the need for greater empirical evidence to advance the construction of a theoretical interpretative framework for the meaning of IAT measures, and also for the meaning of the GV-IAT. Therefore, we consider that providing results related to IPVAW may be an important contribution not only to prevent or reduce IPVAW, but also to support IAT-related research. Moreover, based on this preliminary proposal, new studies could provide more specific evidence on the suitability of GV-IAT, such as the suitability of the stimuli used for the specific measurement of attitudes towards IPVAW in relation to other forms of violence, given the possibility that some subjects may interpret the stimuli related to the category gender violence as examples of violent acts in general, or the words of the category Non gender violence as examples of general non-violent interaction. These results would be of specific interest not only for exploring multi-method forms of measuring attitudes towards IPVAW, but also for exploring the behavior of IAT scores in a new domain that has not yet been explored.

In any case, the discrepancy between implicit and explicit measures cannot be explained solely by factors of social desirability or deliberate attempts at concealment, but rather by the multiple personal, interpersonal, contextual, and methodological moderating factors that are involved (Gawronski & Bodenhausen, 2017; Gawronski & Hahn, 2019; Hofmann et al., 2005; Kurdi et al., 2019; Nosek, 2005). In fact, the processes involved in IAT measures are complex, and despite the progress made in the last two decades, the fundamental debate on the validity of implicit measures in general is still open (Gawronski & Hahn, 2019). Hence, assuming these limitations, it is of the greatest interest to extend the use of IAT measurements to new domains, such as IPVAW, adapting the inferences made to the validity evidence available so far (AERA, APA, & NCME, 2014).

In this regard, recent research suggests that the IAT has limited utility as a measurement method in applied studies or as a measure of individual differences (Tosi et al., 2018); in fact, their most promising use is in a complementary method using the shared variance between IAT scores and explicit measures to control for measurement error in both methods, incorporating a multimethod approach into the measurement of attitudes (Schimmack, 2019). Further studies will be needed to expand sample size and heterogeneity, as well as validity evidence based on the relationship with other variables and response processes (AERA, APA, & NCME, 2014), in order to reach more robust conclusions. All these recommendations can be applied to the GV-IAT proposal presented in this paper.

## Data Availability

The dataset supporting the conclusions of this article is available by request to the corresponding author.

## Appendix

**Table 4:** Target stimuli linked to target category and attribute category (English and original Spanish version)

Categorías	Estímulos
Gender violence (Violencia de género)	Attack (Agredir), Force (Forzar), Humiliate (Humillar), Hit (Pegar), Torture (Torturar), Infringe (Vulnerar)
Non gender violence (No violencia de género)	Support (Apoyar), Collaborate (Colaborar), Cooperate (Cooperar), Empathize (Empatizar), Respect (Respetar), Tolerate (Tolerar)
Good (Bueno)	Wonderful (Maravilloso), Excellent (Excelente), Phenomenal (Fenomenal), Best (Mejor), Positive (Positivo), Optimum (Óptimo)
Bad (Malo)	Horrible (Horrible), Terrible (Terrible), Disastrous (Nefasto), Worst (Peor), Negative (Negativo), Appalling (Pésimo)

## Abbreviations

IAT: Implicit Association Test
GV-IAT: Gender Violence - Implicit Association Test
VAW: Violence against women
IPVAW: Intimate partner violence against women
